# Evaluation of Potential Hormonal Activities of Ashwagandha (*Withania somnifera*)

**DOI:** 10.1002/ptr.70155

**Published:** 2025-12-26

**Authors:** Günter Vollmer, Thomas Brendler

**Affiliations:** ^1^ Fakultät Biologie Technische Universität Dresden Dresden Germany; ^2^ Pharmacognosy Institute, School of Pharmacy University of Illinois Chicago Chicago Illinois USA; ^3^ Department of Botany and Plant Biotechnology University of Johannesburg Johannesburg South Africa

**Keywords:** ashwagandha, endocrine system, HPA axis, HPG axis, kisspeptin, thyroid, *Withania somnifera*, withanolides

## Abstract

Ashwagandha is a widely used herb in traditional medicine systems, particularly Ayurveda. Despite its extensive tradition, growing concerns regarding its potential endocrinological effects have prompted scientific scrutiny. This review systematically evaluates the current preclinical, clinical, and case study evidence concerning AS's effects on hormonal systems, including the thyroid axis, the hypothalamic–pituitary–adrenal (HPA) axis, and the hypothalamic–pituitary–gonadal (HPG) axis. AS appears to elevate thyroid hormones in hypothyroid contexts. Multiple studies consistently show AS‐mediated reductions in cortisol levels, supporting its proposed anti‐stress effects via modulation of HPA‐axis activity. Evidence also indicates AS may influence sex hormone regulation in both sexes, notably increasing testosterone levels in men and affecting estrogen and gonadotrophins in women. Mechanistically, these hormonal effects likely result from modulation of central regulatory pathways rather than direct receptor binding. Limitations in existing studies—such as high dosing in animal models and small sample sizes in clinical trials—underscore the need for more rigorous, dose‐responsive, and mechanistically targeted research. In the meantime, careful consideration is warranted when evaluating AS as a therapeutic agent.

## 1 Introduction

Ashwagandha ( *Withania somnifera* (L.) Dunal, AS) is a widely recognized herb in India's traditional medical systems, collectively known as Ayush, which includes Ayurveda, yoga, naturopathy, Unani, Siddha, and homeopathy. Numerous classical Ayurvedic texts emphasize its broad range of health‐promoting properties, particularly rejuvenating effects and its ability to enhance the body's resilience to stress. In Ayurvedic medicine, the root of AS is classified as a rasayana (rejuvenating tonic), known for supporting weight gain, boosting vitality, and exerting aphrodisiac effects (Balasubramani et al. 2011). Beyond Ayurveda, AS is also used in other medicinal paradigms, underscoring its therapeutic versatility and cultural significance across traditional medicinal practices.

Within the European Union (EU), AS is currently regulated as a food supplement (EU Directorate‐General for Health and Food Safety 2024). However, despite its longstanding use in traditional Ayush medicine, concerns about its safety first emerged in the EU in 2013 (Klenow et al. 2013). Key safety concerns include potential effects on the reproductive system, including a suspected abortifacient action; the impact on thyroid hormones and the broader endocrine system; the inhibition of acetylcholinesterase; and the modulation of immune function.

These issues are compounded by broader questions regarding the maximum safe dietary intake of AS and its safety in vulnerable populations, including the elderly, pregnant women, and breastfeeding mothers. Additionally, there is ongoing scrutiny over whether preparations made from the root, leaves, or combined plant parts of AS vary in their safety profiles (Brendler 2026; Brendler et al. 2026; Williamson and Brendler 2026).

By late 2023, VigiBase—the World Health Organization's (WHO) global repository for individual case safety reports of suspected adverse drug reactions, managed by the Uppsala Monitoring Centre—had logged 15 reports linking AS to hepatobiliary disorders and associated investigations (Lim and Barnes 2024). National regulatory agencies, such as Australia's Therapeutic Goods Administration (TGA), have also recorded adverse events, with most complaints in Australia involving digestive issues, prompting a safety advisory in February 2024 (Therapeutic Goods Administration 2024).

In light of these concerns, the EU Heads of Food Safety Agencies (HoA) working group on food supplements has recommended initiating an Article 8 procedure under Regulation (EC) No 1925/2006 (Heads of Food Safety Agencies 2024). If adopted, this could result in a ban on the use of AS in food supplements. Since AS is not recognized as a traditional herbal medicine under EU law (HMPC 2013), such a ban could effectively lead to its removal from the EU market altogether.

In the following sections, we investigate and evaluate the effects of AS on the endocrine system based on pre‐clinical and clinical evidence. In particular, we focus on the thyroid hormones, hormones of the Hypothalamus‐Pituitary‐Adrenal (HPA) axis, hormones of the female and male reproductive system, which are regulated by the Hypothalamus‐Pituitary‐Gonadal (HPG) axis. We also explore the mechanistic consequences of AS effects on these hormonal systems. This review attempts to address specifically concerns voiced by competent authorities by focusing on the evaluation of safety aspects evolving from efficacy studies.

## 2 Materials and Methods

Anticipating heterogeneity in quality and breadth of the available data, we chose the form of a narrative review, which allowed the analysis to be inclusive and span both pre‐clinical and clinical data. A comprehensive search of standard scientific databases was conducted up to 05/2025 using the following search strategy: (“ashwagandha” OR “ *Withania somnifera* ”) AND ((“endocrine” OR “hormone”) AND (“thyroid” OR “reproductive”)), additional searches were conducted in combination with keywords “withanolides,” “HPA,” “HPG,” “TSH,” “infertility,” “testosterone,” and “cortisol.” References were searched manually. While the primary focus was on preparations derived from AS root, studies using other parts of the plant were also considered where appropriate.

## 3 Results

**FIGURE 1:**
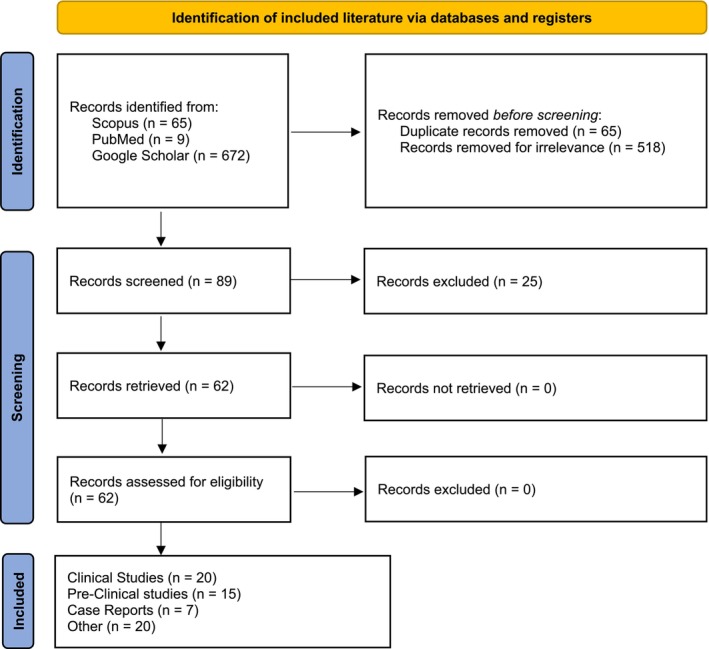
PRISMA flow chart.

Search results are presented in Figure 1.

### 3.1 AS and the Thyroid Hormone System

AS root extract, or its constituent compounds, have been reported to interact with the thyroid hormone system (Javidi et al. 2023; Mikulska et al. 2023). Evidence for this observation stems from three different types of experimental approaches: clinical studies, preclinical animal studies, and case study observations (for summary of hormonal effects see Table 1). It should be noted that only a subset of clinical and preclinical studies specifically aimed to investigate the effects of AS supplementation on the thyroid hormone system. In other studies, thyroid hormone levels were assessed alongside primary outcome measures, often as part of safety evaluations. The aim of this section is to address whether the intake of AS root or root extracts poses a risk to the thyroid hormone system.

**TABLE 1:** Regulation of the thyroid hormone system by AS.

Author	Ashwagandha specification	Dosage/duration	Study type	Hormonal context in study		Hormonal regulation				
				Regulation	Biomarker	T3	fT3	T4	fT4	TSH
Panda and Kar (1997)	Spray dried root powder	333 mg/kg bw/day; 30 days	Animal, cockerel; ♂	X		↑	nd	↑	nd	nd
Panda and Kar (1998)	Aqueous root extract	1400 mg/kg bw/day; 20 day	Animal, mouse; ♂	X		↑	nd	↑	nd	nd
Panda and Kar (1999)	Aqueous root extract	1400 mg/kg bw/day; 20 day	Animal, mouse; ♀	X		nd	nd	↑	nd	nd
Abdel‐Wahhab et al. (2019)	Methanolic root powder extract	500 mg/kg bw/day; 6 weeks	Animal, rat; ♂; hypothyroid	X		↑	↑	↑	↑	↓
Ibrahim et al. (2023)	Extract, plant organ not specified	50 mg/kg bw/day; 30 days	Animal, rat; ♂; hypothyroid	X		↑	nd	↑	nd	nd
Alhahawachee et al. (2023)	Root extract	200 mg/kg bw/day; 21 days	Animal, rat pups			nd	nd	↑	nd	nd
Gannon et al. (2014)	Sensoril standardized extract	250 mg/day; 1 week 2 × 250 mg/day; 7 weeks	Clinical; bipolar patients		X	10 out of 60 patients show unusual thyroid hormone profiles T4↑ (subtle)				
Sharma et al. (2018)	Root extract (Ixoreal)	600 mg/day; 8 weeks	Clinical; hypothyroid	X		↑	nd	↑	nd	↓
Verma et al. (2021)	Root extract (Ixoreal)	2 × 300 mg/day; 8 weeks	Clinical; safety		X	↔	nd	↔	nd	↔
Van Der Hooft et al. (2005)	Herbal extract (Holisan)	1 × 250 mg/day; 6 weeks; 2 × 250 mg complaints	Case report	Thyrotoxicosis		nd	nd	nd	↑	↓↓
Curry et al. (2019)	Root powder and black pepper	1950 mg/3×/week/1 month; 1950 mg/day/2 months	Case report	Thyrotoxicosis		nd	nd	nd	↑	↓↓
Kamal et al. (2022)	Unknown	Unknown/2 years	Case report	Thyrotoxicosis		↔	nd	nd	↔	↓↓
Hayashi et al. (2024)	Unknown	Compliant/2 months	Case report	Thyrotoxicosis		↑	nd	nd	↑	↓↓

Abbreviations: ↑ = up‐regulated, ↓ = down‐regulated, ↓↓ = strongly down‐regulated, ↔ = no change, fT3 = free triiodothyronine, fT4 = free thyroxin, nd = not determined, T3 = triiodothyronine, T4 = thyroxin, TSH = thyroid stimulating hormone.

In studies evaluating the effects of exogenous compounds on the thyroid hormone system, the strength of the study is determined by the selection of the thyroid hormones measured. Studies should assess TSH in combination with one of the free variants of free triiodothyronine (fT3) or free thyroxin (fT4), as TSH is a highly sensitive marker for detecting hypothyroid states. fT3 and/or fT4 levels are more relevant than total triiodothyronine (T3) or thyroxin (T4) levels, as only the free fractions are biologically active. In contrast, the majority of the total hormones are bound to high‐affinity binding proteins and therefore biologically not available (Bikle 2021; Van Uytfanghe et al. 2023).

The direct effect of AS on the thyroid hormone system in humans was studied in only one clinical study. This randomized, double‐blind, placebo‐controlled pilot study included 50 patients diagnosed with subclinical hypothyroidism (elevated TSH, but normal T3 and T4). Participants were randomly assigned to either treatment or a control group. After 8 weeks of treatment, TSH levels decreased toward normal range, and T3 and T4 levels showed a notable increase (Sharma et al. 2018). The effect was found to be time‐dependent, as it was already detectable, though less pronounced, at the study's midpoint during the intermediate hospital visit. Stratification by sex was not performed. These findings show that AS interferes with the thyroid hormone system in patients who exhibit a mild hypothyroid state.

No alterations in thyroid hormone levels were observed in a safety study with healthy patients. In this study, involving a total of 80 participants, thyroid hormone levels were assessed as a safety parameter (Verma et al. 2021). It is difficult to extract meaningful results from this study. The patients had bipolar disorder, and thyroid‐related effects were reported in only 10 individuals with “abnormal” thyroid hormone profiles—three from the AS group and seven from the placebo group. Although this study may not be overly representative for the purpose of this assessment, mild effects on TSH and T4 were observed in the three AS‐treated patients (Gannon et al. 2014).

Thyrotoxicosis describes the clinical outcomes in target organs in response to excess thyroid hormone levels (elevated T3/fT3 and T4/fT4, as well as very low THS levels) (Sharma and Stan 2019). Four case reports describe this condition in patients using AS supplements.

All case reports must be regarded as strong in terms of measurement of thyroid hormones, as all studies assessed TSH and fT4 levels in patients. Although no causal relationship between AS supplement use and the development of thyrotoxicosis could be established, discontinuation of AS consumption in all four cases resulted in effects that suggested an association between the two.

One 32‐year‐old female patient consumed the supplement for 6 weeks (first week 250 mg, thereafter 2 × 250 mg) and presented with chronic fatigue, very low TSH and high fT4 levels, both out of the normal range. Four weeks after discontinuation, hormone levels normalized (Van Der Hooft et al. 2005). The second case report describes a 62‐year‐old female who took an extra strength AS supplement (1950 mg root powder per dose), initially three times per week for 1 month and then daily for approximately another month (Curry et al. 2019). Compared to most AS supplements on the market, the formulation used in this case appears to contain a substantially higher dose. Likely as a result, TSH levels remained very low and fT4 levels high when hormone levels were first assessed 2 weeks after the patient discontinued the supplement. Over time, thyroid hormone levels and other symptoms like changes in the hematocrit returned to normal. The third case report describes a 73‐year‐old female who replaced her levothyroxine treatment, which she had been taking for 2 years to treat hypothyroidism, with AS. Her TSH levels were below the normal range, while fT4 and T3 appeared normal. After discontinuation of AS, the patient returned to the initial hypothyroid state with mildly elevated TSH and mildly lowered fT4 and fT3 levels (Kamal et al. 2022). The fourth case report involves a 47‐year‐old bodybuilder who took AS for 8 weeks (product or dose undisclosed). He exhibited markedly elevated thyroid hormone levels and very low TSH levels, as well as elevated thyroglobulin levels indicative of a hyperthyroidic state (Hayashi et al. 2024).

One further case was reported in an abstract only (Patel et al. 2023). A 27‐year‐old female, taking unspecified amounts of AS extract, presented with unwanted weight gain and extremely elevated DHEA‐S levels. Although the focus was on adrenal gland hormones, thyroid hormones were reported as low TSH and elevated fT4. Following discontinuation of AS, thyroid hormone levels returned within normal range.

A definitive assessment of these case reports is not possible due to the lack of critical information. Specifically, the products consumed were not adequately described. Although a causal relationship between AS supplementation and the development of thyrotoxicosis has not been established, clinicians should remain vigilant for signs of hyperthyroidism in response to AS supplementation.

Several experimental animal studies provide information on the effects of AS intake on the thyroid hormone system. However, similarly to studies in humans, only a limited number have directly assessed the effect of AS on the thyroid hormone system. Others focused on different endpoints but assessed thyroid hormone levels, for example, as safety parameters. Only one study (Abdel‐Wahhab et al. 2019) assessed TSH levels alongside free hormone levels, although some studies chemically induced a hypothyroid state.

Experiments directly assessing the effects of AS on the thyroid hormone system were performed in cockerels. Oral treatment of 15‐day‐old cockerels with 20 mg of root extract led to an increase in T4, while the levels of T3 and the ratio of T3/T4 were not affected (Panda and Kar 1997). The same authors investigated the effects of oral administration of 1400 mg/kg bw/day to male Swiss albino mice on thyroid hormone levels. T4 and T3 levels increased in response to the treatment (Panda and Kar 1998). The only animal study performed exclusively in female organisms was a comparative study on the effects of AS (1400 mg/kg bw/day) and *Bauhinia purpurea* (2.5 mg/kg bw/day) extracts in Swiss female albino mice by gastric intubation. While treatment with *Bauhinia purpurea* extract elevated both T4 and T3 levels, treatment with AS extract only led to elevated T4 levels (Panda and Kar 1999).

Since AS extract has been shown to reverse thyroid hormone disbalances in the case of hypothyroidism in humans (Sharma et al. 2018), another animal study investigating the effects of AS intake on the thyroid hormone system used a hypothyroid experimental animal model. Using a comparative approach, the authors explored the effects of 500 mg/kg bw/day of AS extract in normal and PTU‐treated (chemically‐induced hypothyroid state) albino rats. Interestingly, there were no detectable effects on the levels of TSH, T4, fT4, T3, and fT3 in normal rats, whereas AS treatment normalized thyroid hormone levels in the hypothyroid experimental arm of the experiment by decreasing TSH levels and increasing T4, fT4, T3, and fT3 levels (Abdel‐Wahhab et al. 2019). Models of experimental PTU‐induced hypothyroidism were also used in other studies. One study investigated whether oral administration of 200 mg/kg bw/day of AS extract for 21 days could counteract PTU‐induced hypothyroidism in male albino rat pups (Alhahawachee et al. 2023). Another study investigated the effects of oral administration of 50 mg/kg bw/day of AS extract on the growth plate and the hippocampus of juvenile male albino rats (Ibrahim et al. 2023). Both studies also assessed the impact of treatment on thyroid hormone levels. Oral administration of 200 mg/kg bw/day of AS extract to rat pups led to an increase of T4 although the effect was less pronounced than that observed with the positive control levothyroxine. TSH, considered a more important indicator of hypothyroidism, and T3 were not measured in this study (Alhahawachee et al. 2023). In contrast, oral administration of 50 mg/kg bw/day to hypothyroid juvenile rats decreased TSH and increased T4 and T3, effectively counteracting hypothyroid conditions (Ibrahim et al. 2023).

Another study investigated whether AS extract at 500 mg/kg bw/day for 30 days is capable of attenuating hypothyroidism induced changes in the parotid gland. Unfortunately, no thyroid hormone levels were disclosed in this study (Kashef and Ibrahim 2023).

Thyroid hormones play a critical role in embryonic development and organogenesis. Therefore, potential disruptions to developmental processes caused by compounds impacting the thyroid hormone system are investigated using appropriate developmental animal models, for example, zebrafish. Zebrafish embryos were treated with AS in a dose‐dependent manner, with doses ranging from 1 to 50 μg/mL and administered from 2 to 5 days post fertilization. No developmental disturbances in zebrafish development were observed within the investigated developmental window represented by the treatment period (Naseem et al. 2023).

The studies described above provide preliminary evidence that oral administration of AS extracts can elevate thyroid hormone levels in healthy animals and mitigate thyroid hormone imbalances in chemically induced hypothyroid models in adult and juvenile rodents. However, the relevance of these studies for evaluating efficacy in humans is limited. For example, critical details such as the specific animal species and body weight are often not reported. Moreover, except for the study in zebrafish, none of the rodent studies were performed in a dose‐dependent manner, thereby limiting the potential for proper toxicological assessment. Further, studies are incomplete with respect to key thyroid hormone parameters, as only one study provides values for TSH and fT3 or fT4. Finally, except for two studies, the observations are derived from studies using high pharmacological doses, which are of questionable relevance for humans.

### 3.2 AS and Hormones of the Hypothalamus‐Pituitary Gland‐Adrenal (HPA) Axis

Although no studies have directly examined the potential impact of AS supplementation on adrenal gland function to date, it is known that stress directly affects the hormones within the hormonal cascade of the Hypothalamus‐Pituitary Gland‐Adrenal (HPA) axis. Therefore, research in the context of stress management delivers some insights on the regulation of adrenal gland hormones by AS. Additional insights are provided by studies exploring AS in the treatment of adrenal‐related hormonal imbalances, such as those observed in congenital adrenal hyperplasia (CAH).

Studies investigating effects of AS treatment on stress symptoms, including measurement of cortisol, have been conducted in both clinical and animal models. A summary of hormonal effects within the studies is presented in Table 2.

**TABLE 2:** Regulation of hormones of the adrenal cortex.

Author	Ashwagandha specification	Dosage/duration	Study type	Hormonal context		Hormonal regulation			
				Regulation	Biomarker	CRH	ACTH	Cortisol	DHEA‐S
Priyanka et al. (2020)	KSM‐66 root extract	2, 5, 10 g/animal/day; 21 days	Animal, horse ♀ + ♂		X	nd	nd	**↓**	nd
Dawane et al. (2024)	KSM‐66 root extract	27, 54, 108 mg/kg bw/day; 36 days prophylactic, 21 days therapeutic	Animal, rat ♀ + ♂		X	**↓**	**↓**	**↓**	nd
Chandrasekhar et al. (2012)	KSM‐66 root extract	2 × 300 mg/day, 60 days	Clinical; anti‐stress, anti‐anxiety		X	nd	nd	**↓**	nd
Salve et al. (2019)	KSM‐66 root extract	2 × 125 mg/day or 2 × 300 mg/day; 8 weeks	Clinical, anti‐stress, anti‐anxiety		X	nd	nd	**↓**	nd
Lopresti, Drummond, and Smith (2019) and Lopresti, Smith, et al. (2019)	Shoden root extract	240 mg/day; 60 days	Clinical; stress resistance		X	nd	nd	♂ ↓ ♂ ↓ all ↓	♂↔ ♂↓ all ↓
Majeed et al. (2023)	Root extract ≈ 2.5 mg withanolides	500 mg/day; 60 days	Clinical; anti‐stress, anti‐anxiety		X	nd	nd	**↓**	nd
Kalani et al. (2012)	Not specified	2 × 400 mg/day; 6 months	Case study	Treatment CAH; 3ß‐ol‐dehydrogensae deficiency, aldosterone synthase deficiency		17‐OH‐pregnenolone↓ 18‐hydroxycorticosterone↓ Corticosterone↓ 11‐deoxycortisol↓			
Powell et al. (2017)	Root extract	2 × 400 mg/day 10 months, 2 × 800 mg/day ≈ 15 months	Case study	Treatment CAH; non‐classical 11‐hydroxylase deficiency		11‐doxycortisol↓; time dependent 10 months, 18 months, 3 years			
Fry et al. (2022)	Root extract, British supplements	858.6 mg/day; 10 weeks	Case study	Synacthen test		ACTH response↓			

Abbreviations: ↑ = up‐regulated, ↓ = down‐regulated, ACTH = adrenocorticotropic hormone, CAH = congenital adrenal hyperplasia, CRH = corticotropin releasing hormone, DHEA‐S = dehydroandrosterone sulfate, nd = not determined, OH = hydroxy.

A prospective, randomized, double‐blind, placebo‐controlled study involving 64 participants investigated the effects of twice‐daily supplementation with 300 mg of an AS root extract over 60 days on stress and anxiety. Results showed a reduction in stress and cortisol levels (Chandrasekhar et al. 2012).

A double‐blind, randomized, placebo‐controlled clinical study investigated the adaptogenic and anxiolytic effects of an AS root extract (Salve et al. 2019). Unlike other studies assessing the potential of AS on stress resistance, this study was performed at low and high doses and thus yielded a dose response evaluation. In the study population, 60 participants were allocated to one of three groups: a placebo control group and two treatment groups receiving either 2 × 125 mg extract or 2 × 300 mg extract per day for 8 weeks. Measurements were performed after 4 weeks and at the end of the study. While the primary outcome, a reduction in perceived stress scale (PSS) scores, is not of endocrinological relevance, the study showed that both the low and the high AS dose lowered cortisol levels. This effect, however, was evident only at 8 weeks, with no significant changes observed at the four‐week interim assessment.

Another randomized, double‐blind, placebo‐controlled study investigated the potential anxiolytic effects in adults self‐reporting high stress. According to defined inclusion and exclusion criteria, 60 participants (male and female) were randomized to treatment and placebo groups. Treatment consisted of 240 mg of AS extract per day for 60 days. The study revealed a statistically significant reduction in Hamilton Anxiety Rating Scale and a near statistically significant reduction in Depression, Anxiety, and Stress Scale −21 (Lopresti, Smith, et al. 2019). The second goal of this study was to elucidate the possible mechanism underlying the observed effects of AS supplementation. For this purpose, levels of cortisol, DHEA‐S, and testosterone were measured. AS treatment resulted in a time‐dependent reduction of morning cortisol levels across the overall study population, affecting both females and males. DHEA‐S levels were significantly reduced in females but not in males, with this effect demonstrating a clear time dependency. The authors suggested that the stress‐relieving effects of AS may be mediated through modulation of HPA‐axis activity.

A further randomized, double‐blind, placebo‐controlled clinical study investigated whether AS extract could alleviate stress, anxiety, and improve quality of life (Majeed et al. 2023). In this study, groups of 27 participants received either 500 mg AS extract per day or placebo for 60 days. All measures of stress, anxiety, and quality of life improved. Cortisol was measured in the saliva and not in the blood for unspecified reasons. Serotonin and dopamine were measured in the urine. After 60 days of treatment, morning levels of salivary cortisol decreased, and urinary serotonin levels increased, while evening levels of salivary cortisol and urinary levels of dopamine did not show statistically significant changes. This further supports the finding that downregulation of cortisol contributes to the management of stress and anxiety disorders in response to AS extract.

Adrenocortical production of DHEA‐S and cortisol release were assessed in a study investigating the hormonal and vitality effects of AS supplementation in aging, mildly overweight men (Lopresti, Drummond, and Smith 2019). Participants received 300 mg AS extract twice daily for 16 weeks. The intervention increased DHEA‐S production in the adrenal cortex, but cortisol levels remained unchanged. A more recent randomized, double‐blind, placebo‐controlled study involving 60 participants of both sexes examined the effects of the standardized Shoden extract in the context of stress and anxiety reduction (Mishra and Kumar 2024). Participants were assigned to one of three groups and received either 60 or 120 mg of the Shoden extract or a placebo. Morning cortisol levels were used as one of the primary outcome measures and were found to be reduced in both treatment groups.

The finding that AS treatment leads to downregulation of cortisol levels, a key element in stress management, is also supported by two animal studies.

The first study was conducted in an equine model (Priyanka et al. 2020) to evaluate the potential adaptogenic and immunomodulatory activities of AS root extract. Twenty‐four Kathiawari horses were divided into four experimental groups and treated for 21 days with either a placebo or AS extract at doses of 2.5, 5, and 10 g/animal/day. This dosage is equivalent to approximately 6, 15, and 30 mg/kg bw/day for male horses and 7.2, 18, and 36 mg/kg bw/day for female horses. Each dose led to reduced cortisol and epinephrine levels and elevated serotonin levels. The hormonal changes occurred between day 15 (intermediate measurement) and 21 days (end of study) but were not dose dependent. Notably, this study featured a dose‐dependent design, and the lowest doses fall within the range considered relevant for human equivalent dosing after allometric scaling. Despite this, the overall conclusions are limited by the low effect size and the absence of clear dose‐ or time‐dependency.

The second study, conducted in Wistar rats (Dawane et al. 2024), aimed to investigate the potential anxiolytic and antidepressant effects of an AS root extract, as well as to elucidate the mechanism underlying these physiological effects. The rats were subjected to mild stress and allocated to either a prophylactic or therapeutic group. In the prophylactic arm, rats (*n* = 8; four males, four females) received AS extract at 27, 54, and 108 mg/kg bw/day (reasonable human approximation of dosing) for 36 days, directly after stress induction. In the therapeutic arm, rats were first exposed to stress for 15 days, followed by AS treatment at the same dosages noted above for 21 days. Bother arms included appropriate positive and negative controls. Behavioral assessments demonstrated significant improvement in the anxiety‐ and depression‐like behavior of the AS‐treated animals. Hormonally, AS had a dramatic impact on HPA‐axis activity in both experimental arms. Serum CRH was downregulated by all prophylactic and therapeutic doses of AS, while ACTH was downregulated by all prophylactic and only the highest therapeutic dose of AS. Serum cortisol decreased in the middle and highest prophylactic groups and at the low and high therapeutic dose. However, no clear dose–response pattern emerged for any of the HPA‐axis hormones. The authors also investigated additional hormones and growth factors, reporting that AS supplementation led to a dose‐dependent increase in serotonin and BDNF levels. These findings suggest that suppression of HPA‐axis activity, along with the upregulation of serotonin and BDNF, may partially explain the molecular mechanisms underlying the observed anxiolytic and antidepressive effects of AS in this animal model.

AS has also been investigated in clinical contexts that offer additional insights into its effects on hormones of the HPA‐axis. One such context is congenital adrenal hyperplasia (CAH), which is a group of autosomal recessive genetic defects in cortisol synthesis (van der Claahsen‐ Grinten et al. 2022). Due to intact HPA‐axis function and its feedback mechanism, an accumulation of steroids above the enzymatic blockage occurs, frequently resulting in excess androgen production. In non‐classic or late‐onset CAH, described in two case reports below, enzymatic defects are partial and to a varying degree may be compensated but not restored by HPA‐axis activity (Jha and Turcu 2021). As in CAH, low cortisol levels could also be caused by adrenal insufficiency. A third case also describes whether adrenal insufficiency could be related to AS use.

The first case report on the effects of AS on non‐classical CAH describes a 57‐year‐old woman with 3β‐ol‐dehydrogenase deficiency (Kalani et al. 2012), a form of non‐classical CAH that leads to an excess of precursor hormones within the aldosterone and cortisol biosynthetic pathways. At baseline, the patient had dramatically elevated levels of corticosterone (aldosterone biosynthetic pathway) and 17‐hydroxypregnenolone (cortisol biosynthetic pathway). After approximately 6 months of supplementation with 2 × 400 mg AS root extract, the levels of these two hormonal precursors—in addition to other precursors including 18‐hydroxycorticosterone and 11‐deoxycortisol—were reduced. Overall, these changes suggest that AS root extract may attenuate hormonal dysregulation caused by enzymatic blockages. However, one important aspect not discussed by the authors is the risk of accumulation of 17‐hydroxypregnenolone, as this precursor is also a substrate for the Cyp17‐lyase, leading to elevated DHEA, which in its sulfated version represents an androgen precursor (Auchus 2004; Singh et al. 2022). Elevated testosterone was reportedly measured (though data were not shown) and may explain the patient's scalp hair loss, which improved in the course of the treatment.

A second case report describes a 78‐year‐old woman with a non‐classical 11‐hydroxylase deficiency, who had 2.5‐fold elevation of 11‐deoxycortisol levels (Powell et al. 2017). All other hormonal precursors were within the normal range. Supplementation with 2 × 400 mg AS root extract for 10 months resulted in a reduction of 11‐deoxycortisol to approximately 70% of the pretreatment level. Continued treatment with 1200 mg AS root extract (400 mg in the morning, 800 mg in the evening) led to a further decrease after 18 months (approx. 50% of the pretreatment level) and after 3 years (approx. 36% of the basal level).

A third case report noted AS effects on adrenal gland function in a 41‐year‐old woman initially presenting with generalized bodily pain and cortisol levels slightly below the normal range (Fry et al. 2022). Unknown to the physicians, the patient started taking AS root extract (858.6 mg/d) according to manufacturer instructions around the time of her initial visit. Approximately 3 months later, a short‐term Synacthen test (ACTH stimulation) revealed abnormally low cortisol levels, which aligns with the effects described above. However, application of ACTH during the Synacthen test was unable to stimulate cortisol. AS use completely suppressed the responsiveness of the adrenal cortex to ACTH stimulation, meaning stimulation through the HPA axis, thereby leading to adrenal hypofunction. This effect was reversible upon discontinuation of AS root extract supplementation, but the case underscores a need for caution when evaluating adrenal function in individuals using AS supplements.

A fourth potential case was presented in a meeting abstract (Patel et al. 2023), describing a 27‐year‐old woman with extremely elevated levels of DHEA‐S, a key precursor in testosterone biosynthesis. She had been taking 2 × 600 mg AS extract (product details not specified). Elevated testosterone and fT4 levels were also noted. Six weeks after AS discontinuation, DHEA‐S levels were still elevated but declined by more than 50%, and fT4 levels improved as well; testosterone levels after discontinuation were not reported. Authors concluded that AS supplementation likely disrupted natural hormone physiology.

### 3.3 AS and Women's Health

For a summary of hormonal effects in females see Table 3.

**TABLE 3:** AS and women's health.

Author	Ashwagandha specification	Dosage/duration	Study type	Hormonal context		Hormonal regulation			
				Regulation	Biomarker	LH	FSH	T	E2
Al‐Qarawi et al. (2000)	Self‐collected material	470 mg/kg bw/day; 6 days	Animal, rat♀ 17 and 25 days old		X	↔17 days ↔25 days	↔17 days ↑25 days	nd	nd
Megahd and Gabal (2021)	Root powder (tea extract) (Imtenan, Cairo)	200 mg/kg bw/day; 30 days	Animal, rat♀; 30 days^a^		X	↑	↑	nd	↑
Dongre et al. (2015)	KSM‐66 extract	2 × 300 mg/day; 8 weeks	Clinical, sexual function	na	na	nd	nd	nd	nd
Gopal et al. (2021)	KSM‐66 extract	2 × 300 mg/day; 8 weeks	Clinical, (peri)menopausal health		X	↔	↓	↔	↑^b^
Ajgaonkar et al. (2022)	KSM‐66 extract	2 × 300 mg/day; 8 weeks	Clinical, sexual function and safety	na	na	nd	nd	nd	nd

Abbreviations: ↑ = up‐regulated, ↓ = down‐regulated, ↔ = no change, E2 = 17ß‐estradiol, FSH = follicle stimulating hormone, LH = luteinizing hormone, na = not applicable, nd = not detectable, T = testosterone.

^a^ Compared to oxidatively damaged group, not intact control.

^b^ Sub‐stratified normal weight and overweight.

Two clinical studies describe the improvement of female sexual health and desire following supplementation with 2 × 300 mg of AS root extract for 8 weeks (Ajgaonkar et al. 2022; Dongre et al. 2015). However, the studies relied exclusively on subjective scoring systems without accompanying hormonal biomarkers and are therefore not further considered here.

Another aspect of women's health is that of perimenopausal and menopausal transition. Perimenopause is characterized by irregular secretion of gonadotrophins, which in turn affects the production of steroidal sex hormones. A randomized, double‐blind, placebo‐controlled pilot study investigated the impact of AS root extract (2 × 300 mg daily for 8 weeks) on the well‐being of perimenopausal women (Gopal et al. 2021). Participants receiving AS reported a general improvement in quality of life. Endocrine assessments revealed a statistically significant reduction in serum FSH levels and a corresponding increase in serum estradiol levels. Subgroup analysis showed that this increase occurred in both normal‐weight women and obese women, although basal estradiol levels were much higher in obese women. A statistically non‐significant trend toward decreased LH levels in response to AS was observed, while testosterone remained unchanged. Given that estradiol is a well‐known risk factor for the development and/or the growth promotion of breast cancer, estradiol levels should be carefully monitored in peri‐/postmenopausal women taking AS root extracts.

The potential of AS to modulate gonadotrophin levels is noteworthy and was further investigated in an animal study involving immature female Wistar rats, aged 17 days (before weaning) and 25 days (post weaning) to assess whether AS treatment during early development affects ovarian follicle development and gonadotrophin secretion. The rats were treated for six consecutive days with 470 mg/kg bw/day of an extract prepared from the freeze‐dried inner pulp of AS stems and roots (Abdel‐Magied et al. 2001). In both age groups, LH levels remained unaffected by the treatment. However, in the 25‐day‐old animals, AS extract significantly increased FSH levels, which was accompanied by increased ovarian weight and pronounced folliculogenesis. No treatment effects were observed in the 17‐day‐old animals, and the study did not report levels of sex hormones estradiol and testosterone (a precursor of estradiol). This omission is significant since the studied developmental window represents a critical window for mammary gland development and breast cancer susceptibility in rodents. Previous research has demonstrated that both agrochemicals (Kass et al. 2020) and botanicals with estrogen‐like activity (Boutas et al. 2022; Lamartiniere 2000) can influence these outcomes during early life exposure.

Since injuries of female reproductive organs may cause infertility, authors of another study examined whether AS root extract could mitigate oxidative injury to female reproductive organs. Female Sprague–Dawley rats were administered 1% hydrogen peroxide (H_2_O_2_) in their drinking water (Megahd and Gabal 2021), leading to oxidative damage that affected body weight, ovarian and uterine weight, and reduced estrus cyclicity. In parallel, serum concentrations of female sex hormones LH, FSH, estradiol, and progesterone were also reduced. To assess the potential protective effects of AS, the researchers administered 200 mg/kg bw/day tea extract prepared from AS root for 30 days. Physiological parameters—including organ and body weight as well as estrus cyclicity—improved, and the study reported significant increases in all measured female sex hormone levels (LH, FSH, estradiol, and progesterone). However, by the end of the treatment period, hormone levels in AS‐treated animals remained well below those observed in non‐injured control animals.

### 3.4 AS and Male Reproductive Hormones

Several reviews have examined the potential testosterone‐boosting effects of AS extracts in men and male animal models (Sengupta et al. 2018; Welch et al. 2023; Wiciński et al. 2023). This review also highlights testosterone elevation as a key outcome of AS treatment (see Tables 4, 5, 6). To avoid redundancy, the available data sets were organized into two main subchapters. The first focuses on clinical studies investigating endpoints related to male fertility and sexual health, areas that are generally linked to elevated testosterone levels. The second subchapter addresses a smaller number of studies examining the impact of AS on testosterone in relation to its potential anabolic effects, particularly in older adults or individuals with metabolic disorders. This classification is further justified by distinct mechanistic pathways at the molecular level. The functions related to sexual health are not mediated by testosterone itself but by 5α‐dihydrotestosterone (DHT), a product of 5α‐reductase activity. On the other hand, anabolic functions are directly mediated by testosterone (Handelsman 2020). Beyond evaluating the biological responses to AS treatment, this review also addresses emerging concerns about elevated testosterone levels, which may have previously been overlooked in earlier discussion surrounding the use of AS products.

**TABLE 4:** Regulation of male sex hormones—fertility studies.

Author	Ashwagandha specification	Dosage/duration	Study type	Hormonal context		Hormonal regulation			
				Regulation	Biomarker	LH	FSH	T	E2
Ahmad et al. (2010)	Root powder	5 g/day; 3 months	Clinical ♂^a^, fertility	X	X	↑	↓	↑	nd
Mahdi et al. (2011)	Root powder	5 g/day; 3 months	Clinical ♂^a^, fertility stress	X		↑	↓	↑	nd
Gupta et al. (2013)	Root powder	5 g/day; 3 months	Clinical ♂^a^, analytics		X	↑	↓	↑	nd
Ambiye et al. (2013)	KSM‐66 root extract	3 × 225 mg/day; 12 weeks	Clinical ♂, fertility		X	↑	nd	↑	nd
Chauhan et al. (2022)	KSM‐66 root extract	2 × 300 mg/day; 8 weeks	Clinical ♂, sexual health		X	nd	nd	↑	nd
Mutha et al. (2023)	KSM‐66 root extract	2 × 300 mg/day; 8 weeks	Clinical ♂, erectile dysfunction		X	↔	↔	↑	nd

Abbreviations: ↑ = up‐regulated, ↓ = down‐regulated, ↔ = no change, E2 = 17ß‐estradiol, FSH = follicle stimulating hormone, LH = luteinizing hormone, nd = not detectable, T = testosterone.

^a^ Reported only for the infertile population.

**TABLE 5:** Regulation of male sex hormones—animal studies.

Author	Ashwagandha specification	Dosage/duration	Study type	Hormonal context		Hormonal regulation			
				Regulation	Biomarker	LH	FSH	T	E2
Abdel‐Magied et al. (2001)	Self‐prepared inner pulp stems and root	470 mg/kg bw/day; 6 days	Animal, rat♂		X	↔	↓	↑	nd
Kiasalari et al. (2009)	Root material local market added to food pellets	6250 mg/kg bw/day; 4 weeks	Animal, rat♂		X normal	↑	↓	↑	↔
					X diabetic	↑	↓↓	↑↑	↔
Belal et al. (2012)	Root, freeze‐dried, powdered, added to food pellets	6250 mg/kg bw/day; 4 weeks	Animal, rat ♂		X normal	↑	↓	↑	↔
					X diabetic	↑	↓↓	↑↑	↔
Rahmati et al. (2016)	Grounded root powder into food pellets	5000 mg/kg bw/day; 4 weeks	Animal, rat ♂		X normal	↔	↑	↔	↑
					X addicted	↑↑	↔	↑	↑
Sahin et al. (2016)	Methanol/water extract root (2.5% withanolides)	300 mg/kg bw/day	Animal, rat ♂		X	↑	↓	↑	nd

Abbreviations: ↑ = up‐regulated, ↑↑ = strongly up‐regulated, ↓ = down‐regulated, ↓↓ = strongly down‐regulated, ↔ = no change, E2 = 17ß‐estradiol, FSH = follicle stimulating hormone, LH = luteinizing hormone, nd = not detectable, T = testosterone.

**TABLE 6:** Regulation of male sex hormones—anabolic studies.

Author	Ashwagandha specification	Dosage/duration	Study type	Hormonal context		Hormonal regulation			
				Regulation	Biomarker	LH	FSH	T	E2
Wankhede et al. (2015)	KSM‐66 root extract	2 × 300 mg/; 8 weeks	Clinical ♂; anabolic muscle		X	nd	nd	↑	nd
Lopresti, Drummond, and Smith (2019) and Lopresti, Smith, et al. (2019)	Shoden beads, root extract	2 × 300 mg/day; 16 weeks (cross over approach)	Clinical ♂; fatigue, vigor, salivary levels	X	X	nd	nd	↑	↔
Verma et al. (2023)	KSM‐66 root extract	2 × 300 mg/day; 8 weeks	Clinical ♂ and ♀; anabolic muscle		X male	nd	nd	↑^a^	nd
					X female	nd	nd	↔^a^	nd
Smith et al. (2023)	Root extract Witholytin	2 × 200 mg/day; 12 weeks	Clinical ♂ and ♀; stress, fatigue, hormones	X ♂	X ♂	↑	nd	T (total)	↑
				X ♀	X ♀	↔	nd	T (free)	↑

Abbreviations: ↑ = up‐regulated, ↓ = down‐regulated, ↔ = no change, E2 = 17ß‐estradiol, FSH = follicle stimulating hormone, LH = luteinizing hormone, nd = not detectable, T = testosterone.

^a^ Shown for total and free testosterone.

#### 3.4.1 AS Impact on Male Reproductive Hormones and Fertility

In clinical studies reporting improvements in male fertility following AS treatment, the authors emphasize the role of elevated testosterone levels. Testosterone is produced in the testes by Leydig cells in response to LH secreted by the pituitary gland. Across the available clinical studies, the hormonal profiles and their patterns of regulation present a relatively consistent picture. Importantly, two clinical studies directly compared hormone levels in fertile and infertile men treated with AS. Infertile men had lower testosterone and LH levels along with higher FSH levels compared to fertile men (Gupta et al. 2013; Mahdi et al. 2011). These and two more studies performed with infertile men reported a comparable hormonal regulation pattern to AS treatment (Table 4). Treatment led to increased LH levels, which in turn elevated testosterone levels (Ahmad et al. 2010; Ambiye et al. 2013; Gupta et al. 2013; Mahdi et al. 2011). In three of these studies, except the one by Ambiye et al., FSH levels were reported to decrease following AS treatment. One further study reported an increase in testosterone in response to AS treatment but did not provide data on corresponding pituitary LH and FSH levels (Chauhan et al. 2022). As noted earlier (see section on adrenocortical hormones), a more recent randomized, double‐blind, placebo‐controlled trial investigated the effects of standardized Shoden extract on stress and anxiety (Mishra and Kumar 2024). The study included 60 participants of both sexes across three treatment groups and assessed outcomes in a dose‐dependent manner. Among males, but not females, testosterone levels increased by both 60 and 120 mg doses.

While the effects differed between studies, the overall changes in hormones were modest and remained within normal range for adult males (Table 7). Despite the relatively low sample size across the clinical trials, the findings suggest that AS extracts improve fertility outcomes—an effect likely mediated by increased testosterone levels, which appear to be a key molecular mechanism underlying the biological response.

**TABLE 7:** Regulation of testosterone levels by AS.

Exposure/duration	Species	Testosterone quantification method	% change in response to treatment	Author
470 mg/kg bw/day/6 days	Wistar rats (male)	RIA	58.5 ↓^a^	Abdel‐Magied et al. (2001)
6.25% added to chow/4 weeks	Wistar rats (male)	RIA	47.1 ↑ (normal)^a^ 32.5 ↑ (diabetic)^a^	Kiasalari et al. (2009)
6.25% added to chow/4 weeks	Wistar rats (male)	RIA	33.3 ↑ (normal)^a^ 50 ↑(diabetic)^a^	Belal et al. (2012)
6.25% added to chow/21 days	NMRI rats (male)	ELISA	3 ↑ (normal)^a^ 136 ↑ (addicted)^b^	Rahmati et al. (2016)
300 mg/kg bw/day	Sprague–Dawley rats	ELISA	57.7↑	Sahin et al. (2016)
5 g/day powder/3 months	Human, male	Double antibody RIA	11.5 ↑ Normozoospermic 40.7 ↑ Oligozoospermic 23.8 ↑ Asthenozoospermic	Ahmad et al. (2010)
5 g/day powder/3 months	Human, male	Double antibody RIA	10.3 ↑ normal^a^ 7.6 ↑ normal, smoker^a^ 25 ↑ normal, psychological issues^a^	Mahdi et al. (2011)
5 g/day powder/3 months	Human, male	Double antibody RIA	24.1 ↑ Normozoospermic^a^ 38.8 ↑ Oligozoospermic^a^ 19.2 ↑ Asthenozoospermic^a^	Gupta et al. (2013)
3 × 225 mg/12 weeks	Human, male	Chemiluminescence	17.3 ↑	Ambiye et al. (2013)
2 × 300 mg/8 weeks	Human male	ELISA	17.2 ↑^a^	Wankhede et al. (2015)
2 × 300 mg/8 weeks	Human male	ELISA	13.7 ↑ (saliva)	Lopresti, Drummond, and Smith (2019) and Lopresti, Smith, et al. (2019)
2 × 300 mg/8 weeks	Human, male and female	ELISA	4.6 ↑ (free, male) 1.5 ↑ (free, female) 4.9 ↑ (free, male and female)	Verma et al. (2021)
2 × 300 mg/8 weeks	Human, male	ELISA	16.5 ↑	Chauhan et al. (2022)
60 or 120 mg/60 days	Human, male and female	ELFA	22↑ (60 mg, male) 33↑ (120 mg, male)	Mishra and Kumar (2024)

Abbreviations: Double antibody RIA = double antibody radio immune assay, ELFA = enzyme linked fluorescent assay, ELISA = enzyme linked immunosorbent assay, RIA = radio immune assay.

^a^ No numbers for values given, estimation from bar lengths in graphs.

^b^ Values for total testosterone as given in table are not plausible, therefore not used here.

The available animal studies provide insights into the mechanisms of action of AS but offer little in terms of determining efficacy or safety in humans (Table 5), primarily because important information is not reported. Most notably, the chemical composition of the AS root material, which in most animal studies was self‐prepared, was not characterized and therefore cannot be assessed. Furthermore, the doses administered in these studies are high, raising questions about their relevance in humans. Finally, none of the studies assessed the dose–response relationship, which should be standard in preclinical animal testing.

In total, five studies were identified that investigated the impact of AS exposure on sex hormone levels or alterations in testicular function. All reported data on sex hormone levels following AS treatment. In three of these studies, the pattern of hormonal changes mirrored those observed in clinical studies, showing increases in LH and testosterone levels and decreases in FSH levels. Two of these included healthy and diabetic rats. The rats were exposed to 6250 mg/kg bw/day of self‐prepared AS root powder, incorporated into rat chow (Belal et al. 2012; Kiasalari et al. 2009). Increases in LH and testosterone and a decrease in FSH were reported in both groups, although the hormonal effects were more pronounced in diabetic rats. The third study focused on identifying molecular pathways potentially involved in triggering AS responses, highlighting the Nrf2 and the hemoxigenase‐1 pathways as potential mediators. However, it remains unclear whether they are responsible for the upregulation of LH and testosterone and the downregulation of FSH, findings that align with those of human studies (Sahin et al. 2016).

Other animal studies yielded results less consistent with human data, as shown by an experiment conducted in 20‐day‐old immature male Wistar rats who received 470 mg/kg bw/day of a self‐prepared AS extract from freeze dried root and pulp material for 6 days. Following treatment, testosterone and FSH levels were decreased and LH levels were unchanged; however, the study observed improved testicular parameters and notable spermatogenesis (Abdel‐Magied et al. 2001). Another study aimed primarily to investigate the impact of AS treatment on addiction using an animal model (Rahmati et al. 2016). Rats were exposed to approximately 5000 mg/kg bw/day of AS root powder added to pelleted chow. Sex hormone levels were reported separately for control and addicted animals. In control animals, unlike in other studies, FSH levels increased, while LH and testosterone levels remained unchanged. Interestingly, estradiol levels were elevated. In contrast, addicted animals exhibited increased LH and testosterone, while no changes to FSH levels were observed following AS treatment.

#### 3.4.2 AS Effect on Male Reproductive Hormones and Anabolic Effects

Overcoming infertility is one very important function of male sex hormones, particularly through testosterone‐derived 5α‐dihydrotestosterone. Another important function of testosterone is the regulation of anabolic processes, for example in muscle tissue, but also in other organs such as bone. These anabolic effects have led to the abuse of testosterone to enhance athletic performance (Albano et al. 2021). Due to the effectiveness of male sex hormones on anabolic pathways, their modulation by AS extracts was also investigated in this context.

One study assessed the potential direct anabolic effects of AS extract treatment. In this randomized, placebo‐controlled trial, 57 young adults were assigned to either a treatment or placebo group. Participants in the treatment group received 2 × 300 mg of AS extract per day for 8 weeks and followed a structured muscle resistance training program. The increase in muscle size and strength, as well as the reduction in muscle damage, were assessed as anabolic effects (Wankhede et al. 2015). Although testosterone levels increased significantly from pre‐intervention to post‐intervention within the AS group, these changes did not reach statistical significance when comparing the treatment to the placebo group.

AS treatment was used as an adjuvant to a resistance training program in a clinical study investigating its effects on inflammatory markers. The study included both female and male participants and focused on testosterone serum levels. Importantly, it distinguished between free (active) testosterone and total testosterone. Results showed that free testosterone levels were significantly elevated in males and in the total population of males and females, whereas the increase in free testosterone in females fell just short of statistical significance. There were no significant changes in total testosterone levels observed in response to AS treatment (Verma et al. 2023).

Another study examined the effects of AS on body composition in aging, overweight males. Besides mood state and aging male symptoms, both assessed by questionnaire, steroid profiles of cortisol, DHEA‐S (a marker of adrenocortical hormone status) and the sex hormones testosterone and estradiol were measured in saliva (Lopresti, Drummond, and Smith 2019). DHEA, a well‐known adrenal precursor of both male and female sex hormones, is transported to the gonads in its sulfated form. While DHEA‐S and testosterone levels significantly increased in response to AS treatment, cortisol and estradiol levels remained unchanged. Interestingly, a second measurement taken 8 weeks after the termination of treatment showed that the elevated DHEA‐S and testosterone levels were not sustained.

Sex hormone levels were also reported in a study exploring the efficacy and safety of AS extract in adults experiencing high stress and fatigue (Smith et al. 2023). Participants received 2 × 200 mg of an AS root extract for 12 weeks. Hormonal analysis included measurement of total testosterone, free testosterone, estradiol, LH, DHEA‐S, and TSH. Hormone levels were first compared within each group pre‐ and post‐treatment before assessing differences in hormone levels between groups. In males, testosterone, estradiol, and DHEA‐S levels increased over 12 weeks, while in females, levels of free testosterone and estradiol were found to be elevated. Significant differences between groups were only found in males but not in females. In males, levels of free testosterone and of LH (although only described in the text and not shown in the table) increased in response to AS when compared to placebo.

Given the known relationship between elevated testosterone levels and prostate health risks, two rodent studies explored the potential effects of AS on the weight of the ventral prostate lobe, a particularly androgen‐sensitive target organ. In a comparative evaluation of aphrodisiac activities of herbal extracts in male rats, no impact in ventral prostate weight was reported in response to 300 mg/kg bw/day of AS for 8 weeks. Similarly, the weights of the seminal vesicles and the epididymis remained unchanged (Sahin et al. 2016). In contrast, a separate dose‐dependent animal study (100, 200, and 400 mg/kg bw/day) in albino rats reported that prostate weight significantly increased compared to control at the two higher doses (Ganu et al. 2010).

## 4 Discussion

Our review investigated all documented effects of AS on the endocrine system stemming from both pre‐clinical and clinical research. Limitations lie primarily in data availability and quality.

First, we addressed the assumption that AS extracts may be useful to treat hypothyroidism (Wiciński et al. 2023). Observations from animal studies support this claim, although mostly at very high doses. AS treatment in humans may lead to elevated thyroid hormone levels, as four case studies reported symptoms of thyrotoxicosis upon AS intake. Overall, evidence regarding potential effects of AS treatment on the thyroid hormone system remains weak. Animal studies were not performed dose‐dependently and only at high pharmacological doses. Further, levels of TSH and fT3/fT4 were not measured in parallel. The human studies performed thus far are pilot in nature, with relatively low sample sizes. Therefore, until more robust evidence is available, caution may be advisable, particularly regarding the potential risk of thyrotoxicosis.

In anti‐stress and anti‐anxiety studies, AS treatment has consistently been associated with a reduction in cortisol levels. One study reported the downregulation of the adrenal precursor hormone DHEA‐S, while others observed a decrease in epinephrin and an increase in serotonin levels. Collectively, these findings suggest that lowering cortisol levels represents a key element regarding the mediation of potential anti‐stress effects of AS root extracts, supported by the regulation of additional hormones involved in the stress response. Mechanistically, the results of both clinical and animal studies suggest that AS treatment acts on the HPA‐axis, resulting in suppressed cortisol production. However, these studies are based on studies with small sample sizes, limiting the robustness of the evidence. Three case studies have reported on the role of AS in regulating the biosynthesis of adrenal gland hormones, with a particular focus on cortisol. Each provides evidence that the observed effects of AS are mediated through the modulation of the HPA‐axis. In two cases, this mechanism was associated with an improvement in hormonal imbalances linked to non‐classical CAH. In one case study, AS was found to inhibit the expected cortisol response to ACTH in the Synacthen test, a diagnostic tool used to assess adrenal function. If this effect reflects a general pharmacological property of AS, its interference with the Synacthen test may compromise the detection of adrenal hypofunction.

Studies assessing female sex hormone levels in response to AS treatment have primarily focused on the potential impact of AS on female sexual and reproductive health or menopausal health. The low number of studies does not permit firm conclusions on whether AS treatment is useful in the treatment of conditions of female health. However, alterations in pituitary‐derived gonadotrophins levels suggest that effects observed are mediated through modulation of the HPG‐axis.

In males, treatment with AS extracts improved sexual health and fertility, often accompanied by an increase in testosterone levels. The reported changes in pituitary‐derived gonadotrophins suggest that the effect of AS on testosterone levels is also regulated through the HPG‐axis. Although AS treatment was administered in extraordinarily high doses in animal studies that do not correspond to the ranges typically used in humans, findings corroborate clinical data indicating that AS modulates hormone levels through the HPG‐axis.

Several studies in humans and rodents report an improvement of male sexual health and fertility in response to the treatment with AS extracts. The testosterone‐boosting activity of AS root extracts appears to be a key factor contributing to these outcomes. Depending on the study, the overall increase of testosterone levels measured in serum or saliva in males ranges from 3% to 58% (Table 7), with only one study in addicted rodents reporting an increase exceeding 100%. Not all publications disclosed values for the measured testosterone levels in the main text. In these cases, the bar lengths in figures from these publications were measured and used to estimate the effect size, expressed as percentage change relative to the control group in response to treatment. Across studies, baseline testosterone levels were well within the normal range in humans and rodents. Importantly, the increased testosterone levels following AS treatment remained within the normal clinical ranges, indicating that the biological responses were triggered by increases in testosterone levels that, while elevated, remained normal.

It is proposed and accepted that AS supplementation may increase testosterone levels; however, within the normal physiological range, it may improve male fertility and/or muscular strength. Despite its anabolic properties, AS root extract is not currently listed as a prohibited substance by the World Antidoping Association (WADA), even though this list contains a sub‐chapter on “Hormone and Metabolic Modulators.”

Given the testosterone boosting activity of AS, it is reasonable to question whether these increases could also affect other testosterone‐sensitive organs, thereby posing a potential risk for cardiovascular disease (Yeap et al. 2024), prostate cancer in men or PCOS in women (Lopresti, Drummond, and Smith 2019), particularly in predisposed populations (Barata et al. 2024). Given that AS treatment may increase prostate weight and to reduce a potential risk of effects on prostate cancer or precancerous lesions, more comprehensive (pre)clinical studies into effects of AS on prostate health are warranted. A similar need exists for PCOS‐related studies in females. Concerning cardiovascular effects, current evidence suggests that AS may exert a protective effect, particularly through its anti‐inflammatory activities (Wiciński et al. 2023).

Finally, we address the modification of sex hormone levels in response to AS, which is not only detectable on the level of gonadal hormones in males and females but also in the corresponding pituitary gland gonadotrophins, from a mechanistic perspective. This requires a discussion of the potential molecular mode of action of AS extracts. As described above, the regulation of the level of adrenocortical hormones appears to follow similar principles to those governing sex hormones.

Neither extracts nor their individual compounds appear to act through the corresponding nuclear steroid hormone receptor. Consequently, a direct, receptor‐mediated effect in a target cell of individual constituents of AS extracts can be excluded. Instead, the mechanisms seem to be indirect, increasing endogenous hormone levels through regulation of the hormonal axes. Applied to sex hormones, AS not only regulates the levels of testosterone and estradiol but also the corresponding pituitary gland hormones LH and FSH. Likewise, with regard to the hormones of the HPA‐axis, regulation extends beyond cortisol to include hypothalamic CRH and pituitary gland‐derived ACTH, as demonstrated in at least one case study. Taken together, these observations suggest the involvement of the HPG‐ and the HPA‐axis in the regulation of the target hormones testosterone, estradiol, DHEA‐S, and cortisol.

Secretion of hormones of the HPG‐ and HPA‐axis is driven by a pulse generator (Herbison 2018, 2020), which generates pulses at intervals ranging from approximately 90 min to several hours. The amount of the hypothalamic hormones GnRH and CRH, which are secreted into the blood system, primarily depends on the frequency of these pulses and secondarily on their amplitude. The molecular mechanism of the female GnRH pulse generator is the best‐studied example of these kinds of “pacemakers” and is therefore used here as an example.

To understand the mechanism of pulsatile hormone secretion, one must consider the elements constituting the pulse generator. In the case of sex hormones, GnRH releasing cells ultimately trigger the cascade of hormonal events within the PPG‐axis. In humans, GnRH releasing cells are located in the preoptic area and in the infundibular nucleus of the hypothalamus, whereas they reside in the anteroventral periventricular nucleus and in the arcuate nucleus of rodents (Skorupskaite et al. 2014). Pulsatile GnRH secretion is regulated by kisspeptin, a peptide hormone produced by kisspeptin neurons. Kisspeptin neurons in the infundibular (humans) nucleus co‐express neurokinin B and dynorphin and are termed KNDy neurons. Neurokinin B via neurokinin B receptor and dynorphin via kappa opioid peptide receptor autosynaptically regulate pulsatile kisspeptin secretion, with neurokinin B being the stimulatory and dynorphin the inhibitory principle (Moore et al. 2018; Skorupskaite et al. 2014). From this, we hypothesize that AS extract, or its constituents, exert their hormone stimulatory effects by modulating the pulse generator system. This hypothesis is supported by the observation that in addition to estradiol and progesterone, which regulate pulse generation via the well‐known negative feedback mechanisms, a variety of other factors, including metabolic hormones, impact pulse generation (Spaziani et al. 2021). Among these are GABAergic activities, which are also mediated by AS (Murthy et al. 2022; Park et al. 2023). This represents one potential pathway through which AS could influence the pulse generator system. Whether other mechanisms exist eventually leading to modulation of the kisspeptin system remains unknown. For AS, or its constituents, to be able to act within the hypothalamus, they must be able to cross the blood‐brain barrier, an ability that has been predicted for withanolides (Modi et al. 2022; Silva et al. 2025) and demonstrated for withanamides (Vareed et al. 2014). Overall, investigations into the molecular mode of action of AS, particularly regarding interference with the pulse generator, are of significant pharmacological and endocrinological interest, as they may uncover new drug targets or lead to the development of new therapeutics.

## 5 Conclusion

The reviewed data reveal clear limitations. Most animal studies lacked state‐of‐the‐art, dose‐dependent design and used very high doses, limiting their relevance to humans. Nonetheless, pharmacological dosing results may still inform the molecular mechanisms of AS.

Many human trials were randomized and controlled but remain preliminary due to small sample sizes and, in some cases, restricted participant selection (e.g., infertile men only). Inclusion of healthy controls would strengthen future studies.

Reported adverse events are rare relative to the sharp rise in AS use but warrant continued monitoring and appropriate warnings until causality is clarified. Individual case reports should not define AS's overall risk–benefit profile.

Mass spectrometry now supersedes antibody‐based assays in hormone analysis, offering greater precision and the ability to quantify multiple metabolites—especially valuable for matrices like saliva.

AS appears to modulate hormone systems via the hypothalamic–pituitary–gland axes, increasing thyroid and sex hormone levels and lowering cortisol. While elevated thyroid hormone may aid mild hypothyroidism, reports of thyrotoxicosis warrant caution. Reduced cortisol likely underlies AS's anti‐stress effects, though potential adrenal interference requires study.

AS's influence on female hormones, fertility, and menopausal symptoms is promising but inconclusive. In men and women, testosterone tends to increase within normal ranges, correlating with improved fertility and muscle strength, though possible adverse effects (e.g., prostate cancer or PCOS risk) remain underexplored.

Understanding AS's molecular action—potentially involving GnRH and kisspeptin pathways—could uncover new therapeutic strategies for hormone regulation. However, these mechanisms remain largely unexplored and should be prioritized in future research.

## Author Contributions

Günter Vollmer and Thomas Brendler contributed equally to this publication.

## Funding

The authors have nothing to report.

## Conflicts of Interest

The authors declare no conflicts of interest.

## Data Availability

Data sharing not applicable to this article as no datasets were generated or analyzed during the current study.
